# A pharmacist-led follow-up program for patients with coronary heart disease in North Norway–a qualitative study exploring patient experiences

**DOI:** 10.1186/1756-0500-7-197

**Published:** 2014-03-29

**Authors:** Beate Hennie Garcia, Sissel Lisa Storli, Lars Småbrekke

**Affiliations:** 1 Hospital Pharmacy of North Norway Trust, PO 6147, Langnes, 9291 Tromsø, Norway; 2 Department of Health and Care Sciences, Faculty of Health Sciences, University of Tromsø, N-9037 Tromsø, Norway; 3 Department of Pharmacy, Faculty of Health Sciences, University of Tromsø, N-9037 Tromsø, Norway

**Keywords:** Qualitative study, Patient experience, Follow-up program, Clinical pharmacist, Coronary heart disease

## Abstract

**Background:**

Coronary heart disease (CHD) is one of the leading causes of death worldwide. Scientific literature shows that prevention of CHD is inadequate. The clinical pharmacist’s role in patient-centred care has been shown favourable in a large amount of studies, also in relation to reduction of risk factors related to CHD. We developed and piloted a pharmacist-led follow-up program for patients with established CHD after hospital discharge from a hospital in North Norway. The aim of the present study was to explore how participants in the follow-up program experienced the program with regard to four main topics; medication knowledge, feeling of safety and comfort with medications, the functionality of the program and the clinical pharmacist’s role in the interdisciplinary team.

**Methods:**

We performed semi-structured thematic interviews with four patients included in the program. After verbatim transcribing, we analysed the interviews using “qualitative content analyses” by Graneheim and Lundman. Trial registration http://www.clinicaltrials.gov: NCT01131715.

**Results:**

All participants appreciated the follow-up program because their medication knowledge had increased, participation had made them feel safe, they were reassured about the appropriateness of their medications, and they had become more involved in their own medication. The participants reported that the program was well structured and the clinical pharmacist was said to be an important caretaker in the health-care system. The importance of collaboration between pharmacists and physicians, both in hospital and primary care, was emphasized.

**Conclusion:**

Our results indicate that the follow-up program was highly appreciated among the four participants included in this study. The results must be interpreted in the context of the health care system in Norway today. Here, few pharmacists are working in hospitals or in close relation to the general practitioners. In addition, physicians are short of time in order to supply appropriate medication information, both in hospital and primary care. Involving pharmacists in follow-up of patients with CHD seems to be highly appreciated among patients and may be a step towards improving patient care. The study is limited by the low number of participants.

## Background

Coronary heart disease (CHD) is one of the leading causes of death world-wide [1]. A wealth of scientific evidence shows that lifestyle interventions, control of risk factors and use of cardioprotective drug therapies can reduce the risk of recurrent nonfatal and fatal disease and improve the chances of survival [2]. Despite this, Kotseva *et al*. has during three cross-European surveys (EUROASPIRE I-III) showed that secondary prevention of CHD is alarmingly inadequate with persistent smoking habits, high prevalence of obesity, inadequate control of blood pressure, lipids and blood glucose, with most patients not achieving therapy guideline defined targets [3]. In the wake of the EUROASPIRE surveys, a nurse-coordinated follow-up program for patients with CHD (EUROACTION) was initiated and rolled out as a randomized controlled trial (RCT) in eight European countries. Interpretations from the RCT include that local preventive cardiology programs adapted to individual countries is needed in order to care for coronary and high-risk patients [4].

A growing body of studies now indicates that involvement of the clinical pharmacist in direct patient care improves medical therapy and patient outcomes relevant to secondary prevention of CHD, for example blood pressure, blood lipids, prescription of cardioprotective drugs [5-10]. Despite this, few follow-up programs for patients with CHD described in literature, involve the clinical pharmacist as a one of the main stakeholders. Taveira *et al*. describes a Pharmacist-Led Cardiac Risk Reduction Clinic (CRCC) in the USA, where risk reduction in terms of Framingham risk score for CHD was reduced in patients participating in the CRCC [11,12]. Reilly and Cavanagh describe a successful pharmacist-nurse-run clinic for patients with cardiovascular disease in terms of optimizing drug and lifestyle therapy [13]. Lee *et al*. showed positive effect of a pharmacy care program (FAME) in terms of increased medication adherence, medication persistence and clinically meaningful reductions in blood pressure [14].

At the University Hospital in North Norway, a new follow-up program for patients with established CHD was developed and piloted during 2009-2011 (unpublished data). The program was run by a clinical pharmacist. The intervention group receiving follow-up finally included 49 patients. Few studies have explored patient experience with pharmacist involvement in patient care, though high satisfaction have been observed [15-20]. We aimed to explore the experience of participating patients in the follow-up program. We chose to use a qualitative approach with semi-structured interviews, because questionnaires do not widely allow for elaboration of answers. The study was performed by an undergraduate masters student in Pharmacy, and formed basis for his masters thesis in 2010 [21].

## Methods

### The follow-up program

Only patients with established CHD were included, meaning patients who had a diagnosis of myocardial infarction, angina pectoris, coronary artery bypass graft operation or coronary stent implantation. During a twelve-month period after discharge from the Department of Cardiology, the clinical pharmacist had consultations with patients three times; at discharge (at the ward), after three and twelve months (at the hospital pharmacy). The meetings lasted 30-60 minutes and comprised mediation reconciliation, medication therapy review, and patient education concerning medications and lifestyle (physical activity, smoking cessation and heart-friendly diet). Identified drug related problems were communicated to the patients’ physician either personally (first meeting) or by phone or letter (the two last meetings). After each meeting, patients received a written summary from the meeting in addition to laboratory results, correct medication list, information about the specific medications they were using, and individual instructions. The general practitioner (GP) received a letter with laboratory values, identified drug-related problems and recommendations on how to handle these.

### Enrolment of participants

Participants in the follow-up program that had met with the clinical pharmacist at least twice were eligible for inclusion if they were living within the city boundary of Tromsø. Because of the limited time available for the masters project, we aimed to include at least four to six participants. In the period February-March 2010 the clinical pharmacist handed out written information about the study to eligible patients at the end of the consultation. To conceal patient participation for the pharmacist, the patients were asked to read the information at home and to return the signed consent paper to a third party by normal mail. A pre-paid envelope was supplied. To avoid including more than the estimated number of participants, the pharmacist recruited patients in blocks of four. If a sufficient number was not included after one recruitment block, the pharmacist was asked to repeat the procedure. The enrolment procedure was repeated four times in total, ending in March 2010 when five patients had returned their consent papers. These patients were contacted by phone and a face-to-face interview was arranged. Participants were free to choose whether the interview should be held in their home or at the Department of Pharmacy (close to the hospital). One participant withdrew before the interview because of sudden illness. Finally, two males and two females were interviewed. They were aged 47, 57, 62 and 71 years.

### The interviews

A semi-structured interview guide was developed based on four main topics:

•How did the follow-up program influence patient knowledge about medicines?

•How did the follow-up program influence the patients’ feeling of safety and comfort with medication?

•How was the functionality of the follow-up program?

•What did the participants think about the clinical pharmacist’s role in the interdisciplinary health-care team?

To begin the interviews, participants were asked the question: *“Can you tell me about the follow-up you are receiving from the clinical pharmacist?”* Participants were encouraged to speak freely, in order to capture the narrative. The interviewer asked questions rooted in the main topics to elaborate, clarify and confirm understanding.

Interviews were held during February and March 2010. None of the interviewees chose to perform the interviews at home. Interviews lasted 40-60 minutes, were audiotaped and transcribed verbatim by the interviewer directly afterwards. Field notes were taken. To ensure that both the manifest and the latent content were reflected in the narratives, the interviews were listened to several times during the transcription phase. The narratives were subsequently “washed”, meaning that all sentences were structured to make sense and extra words were omitted. Quotes were subsequently translated into English by the principal investigator (BHG). Translation was verified by the co-authors. To give feedback on interviewing technique and content, the whole research group read thoroughly through the first narrative before the next interview was held. The whole research group read all interviews before data analysis.

### Data analysis

An initial analysis was performed by the master student using the thematic approach described as ‘qualitative content analysis’ by Graneheim and Lundman [22]. This approach comprises the following elements: i) reading of all texts several times in order to obtain a sense of the whole; ii) identifying *units of analysis* in accordance with the main topics and bringing these into texts; iii) identifying *meaning units* and abstracting these into *condensed meaning units*; iv) labelling the condensed meaning units with a *code*; v) comparing the codes concerning similarities and differences and sorting them into *main-categories* and *sub-categories*, which constitute both the manifest and the latent content of the interviews, vi) summarizing the contents of the main categories to generalized descriptions and experiences reflecting the most important aspects of each topic in the interview guideline. The same analysis was subsequently made by the principal investigator (BHG), and validated by the research team (SLS, LS), who agreed upon the final interpretation.

### Ethics

This study has been conducted in accordance with the principles of the Helsinki Declaration [23]. Informed consents were obtained from all participants and the Regional Committee for Medical Research Ethics North Norway approved the study. Registration number at http://www.clinicaltrials.gov is NCT01131715.

## Results

The four initial main topics were eventually reduced to three topics. The topic ‘feeling of safety and comfort with medication’ was merged with the topic ‘experiences with the follow-up program’, based on the finding that all four participants expressed that the program itself had made their ‘feeling of safety and comfort with medications’ increase. In Table 1, the findings from the analysis are shown. In the following sections, condensed versions of the three final topics are given. The informants are re-named Steve (62 years) , Bryan (47 years), Claire (57 years) and Mary (71 years).

**Table 1:** Results from ‘qualitative content analysis’ of four narratives; topics, main-categories and sub-categories

**Topic**	**Main-categories**	**Sub-categories**
Experiences with the follow-up program	Abstract	Feelings of safety
		Personal opinions about the pharmacist and the framework
	Specific	Medication
		Execution
		Clinical pharmacist performance
Knowledge about medications	Insight	Use
		Importance
		Medication properties
	Attitude towards medications	Adherence
		Concordance
The clinical pharmacist’s role in the interdisciplinary team	Primary care	General practitioner
		Clinical pharmacist
		Community pharmacies
	Hospital care	Hospital ward
		Heart school
	Ideal collaboration	Clinical pharmacist task
		Overall

### Experiences with the follow-up program

Experiences with the follow-up program were found to be characterized by its framework and execution, the pharmacist’s performance and her empathic behaviour. In addition, all participants said that the program had made them feel more safe and comfortable with their medications. These experiences were categorized into ‘specific’ and ‘abstract’ experiences, the latter category covering feelings and personal opinions.

The informants described the follow-up program with words like ‘great’, ‘a positive experience’, ‘educative’, that they felt ‘safeguarded’ and ‘fortunate’, that they had ‘received special treatment’ and ‘help in daily life’. All four informants stated that the follow-up program should be recommended to everybody that uses medications. Steve suggested that everybody using medications should be offered pharmacist counselling at least once per year. Claire described it like this:

“You don’t get enough information about medicines. So, you’ve really made a discovery with this program! Everybody using medications, or at least everybody who use medications after hospitalization, and who has to use medications life-long, should have this kind of follow-up” (Claire) [1]

All participants expressed that they had gained new and clarifying information about their medication. Claire, Mary and Bryan even believed that their medication regime had become better and more appropriate. All participants appreciated the pharmacist’s thorough knowledge about medications and her critical attitude towards them, which can be explained by the general scepticism towards medications expressed by all four participants. In fact, It seemed like they most of all did *not want* to use medications, or at least wanted to keep them at a minimum. In addition to the pharmacist’s professional skills, the emphatic way of acting with patients played an important role when educating about medications. Mary said:

“She was a human being that I could talk to. One that was professionally educated, who was skilled in supervising me about my medications and my disease” (Mary) [2]

How the follow-up program was carried out seemed important to the participants. The following elements were highlighted: the generous time schedule during follow-up, the proximity to the place where meetings took place, and the fact that they received summary letters and copies of correspondence with the GP.

Bryan and Steve also preferred personal meetings over phone meetings. They said that it established a closer relationship with the pharmacist and that they could devote themselves fully to the follow-up issues without being disturbed. Mary and Bryan also felt that the follow-up program had been presented to them in a respectful way, not *‘like a salesman’* as Bryan stated it. Claire, Bryan and Mary also appreciated that they did not have to keep track on so many things during follow-up, as Bryan announced:

“If I would have to write a diary, journal or remember anything in particular, like sending in reports or whatever… I doubt that I would have joined–I’m so bad in keeping track of such things”. (Bryan) [3]

All four informants said that they felt safe when being part of the follow-up program. The feeling of safety seemed to be related to both the pharmacist’s professional skills as well as the predictability of the follow-up program. The fact that the pharmacist supplied a ‘second opinion’ about the medications, the availability of the pharmacist by phone, and the honest answers to questions also seemed to contribute to the feeling of being cared for. Consequently, this made participants feel safe. That the participants felt safe while participating in this program seemed to be related to their feeling of *not* being properly taken care of by the public health care system. Claire said:

“If I had not joined this program, I would have been all on my own” (Claire) [4]

### Knowledge about medications

All participants said that they had gained knowledge about medications in some way or another. They had increased their knowledge within the following topics: ‘medication use’, ‘how to take medications’ and ‘their own attitude towards medicines’. The latter category could be dichotomized into the well-known principles within pharmacy; ‘adherence’ and ‘concordance’, respectively reflecting ‘The extent to which the patient’s behaviour matches agreed recommendations from the prescriber’ and ‘a consultation process, in which doctor and patient agree upon therapeutic decisions that incorporate their respective views’ [24,25].

Knowledge gained by participants seemed to be founded in the thorough and comprehensible information given by the pharmacist, which was highlighted by all informants. Mary stated:

“She explained in a manner so that I understood like I’d never done before” (Mary) [5]

This shows the importance of communication skills and communicating with patients in a language they understand. Claire said she had become more adherent with her medication regime, as she now recognized the importance of her medications. Steve and Bryan said they had become more conscious about their medication use, which indicates a higher understanding, which is one of the cornerstones of ‘the concordance principle’. Everybody expressed that they had acquired specific knowledge, either about the correct time of the day to take medications (for example simvastatin at night in order to obtain optimal effect), important food-drug interactions (for example calcium absorption may be inhibited by dietary fibre) or the drugs’ intended effect (for example β-blockers to slow your heart rate). Claire and Steve also felt they had acquired knowledge about side effects (for example that ‘aspirin may be hard for your stomach’ and ‘simvastatin may give you muscle pain’). For Bryan, the information from the clinical pharmacist had made him aware that he actually *had* side effects from his β-blocker:

“And it struck me as a lightening, the cause of all my troubles. They were side effects of the medications I was using, sweating, headache, no energy, heart rate under 40 and very lazy…” (Bryan) [6]

Bryan also told that he previously took his medications because his doctor told him so. The other participants indicated similar attitudes, which is the opposite of the ‘concordance principle’. The fact that they all emphasized how important knowledge about medication was indicates that they were pleased with approaching ‘concordance’. Mary said:

“It’s positive to know more about what you put in your mouth and swallow with water… and believe that this helps for this and that. But to know *why* it helps and *how…* that’s what I think is positive. That you learn more about it, not just eat and swallow what the doctor has told you to.” (Mary) [7]

### The clinical pharmacist’s role in the interdisciplinary team

The participants’ experiences with the pharmacist as part of the interdisciplinary team, was frequently expressed in comparison with their experiences with the public healthcare service in general; the GP, the hospital and the community pharmacies. Our findings have to be seen in the light of the Norwegian healthcare system, where patients are not familiar with encountering clinical pharmacists. Consequently, they compare their experience with the follow-up program with the healthcare service they normally receive.

All four participants expressed that time with and information from their GP was inadequate. They said that the pharmacist had been more available for questions. Bryan called the GP system an ‘on-going industry’. His argument was that that he always exited the physician’s office within ten minutes after entering, including payment, feeling that the consultation is too short. Mary thought that explanations she received from her physicians were of poor quality. In fact, when the participants spoke about their GPs, they all expressed feelings like ‘lack of confidence’, ‘not being taken seriously’ and ‘given a prescription to end the appointment’. Seen in the light of such experience, it is perhaps not strange that they appreciated the pharmacist’s suggestions medication amendments, and that they acknowledged the pharmacist as part of the interdisciplinary team as the one offering a ‘second opinion’ of their medications. Steve said:

“I think it’s reasonable that a pharmacist has an overview parallel to the physician’s, and perhaps also has a different view on things” (Steve) [8]

Bryan, Claire and Mary told that their GPs had appreciated and welcomed the pharmacist’s medication recommendations. Mary said that she had actually feared a negative reaction from her GP, and indicated that GPs might experience pharmacist involvement as interfering with their area of expertise.

The participants regarded community pharmacies like more or less “normal shops”, where ‘you hand in your prescription, you pay and you leave’, as Bryan stated. Claire said that the follow-up from the clinical pharmacist was something totally different and that she had never experienced to get information from pharmacists in a community pharmacy. She now wanted the community pharmacists to be more available and visible in the pharmacies. Steve also felt that it would be easier to approach community pharmacists after the follow-up program.

The clinical pharmacist’s role in the hospital service was acknowledged by all participants, and must also be seen in relation with their disappointment concerning follow-up from their hospital. Involvement from a pharmacist at the ward was regarded as positive because of the pharmacists’ skills within medication counselling. Again, this must be seen in relation to the physicians’ shortage of time. Mary said:

“I think that having a pharmacist at the hospital ward would be very good. They can explain about medication use, because the doctors, unfortunately, don’t have time for that” (Mary) [9]

Steve also had thoughts about ‘the Heart School’, which is a two-day follow-up program offered by the University Hospital of North Norway to patients that have experienced their first myocardial infarction. The program comprises group teaching by a cardiologist about CHD, by a pharmacist about medications, by a physiotherapist about physical activity and by a nutritionist about diet and heart-protective food. The patients also receive a one-to-one consultation with the cardiologist. Steve suggested that the pharmacist should also perform one-to-one consultations, in the same manner as the cardiologist. He said:

“You have the physiotherapist and the expert in nutrition. You have the cardiologist, and you have the pharmacist. Those four are important, I believe… and I’ve experienced that, at the Heart School…. Those four should form a team. The result will be a better understanding of the relationship between medications, diet, physical activity and stress. [….] It’s important that the patients with coronary heart disease get to speak with the pharmacist. This may be feasible at the Heart School. Time for personal consultations with the pharmacist *must* be a priority” (Steve) [10]

Altogether, pharmacist collaboration with the GP and the hospital physician were appreciated and seen as important, both to inform about mediation, and to perform ‘quality checks’ of medication regimens. Claire thought that all patients at a cardiology ward should meet the pharmacist before discharge. Steve was also convinced about the economy in this; he thought this could make a means to get patients more rapidly back to work. Concerning medication dispensing at the community pharmacy, Steve found it troublesome if he had to return to his GP for a new prescription because the pharmacist revealed a drug related problem. This indicates that medication therapy review should be performed at the time of prescription.

Despite the positive endorsement of the pharmacists’ role in the interdisciplinary health-care team, there were concerns raised by the participants regarding how different tasks should be distributed between the different members of the healthcare team. In addition, the concern for the patient having too many healthcare professional to relate to was also mentioned. Steve said:

“I believe the patients will benefit from the collaboration between the physician, the pharmacist and the nurse–if it’s done properly. However, the patient cannot relate with too many persons. It might not be necessary for the pharmacist to have their own consultations with the patients at the ward, but the physician must use the pharmacists’ knowledge more than today… I’m sure they [the pharmacists] have more knowledge about medicines than the physicians. But the specific way to go forward can be troublesome… to find the middle way.” (Steve) [11]

## Discussion

Applying a thematic approach, this study is the first study to explore experiences from patients with established CHD with a pharmacist-led one-year follow-up program after hospital discharge. All participants highly appreciated the program, which is in accordance with findings from other studies where satisfaction with pharmacist involvement in direct patient care has been explored [15,17,19]. Time seemed to be an important factor for all participants, which must be seen in relation to their experience of shortage of time by healthcare professionals in general. Tsiantou *et al*. also found that time spent by pharmacist in patient relationships was as an important factor in order to increase medication adherence [18].

But time was not the only factor that contributed to the participants’ positive experience with the follow-up program. As also mentioned by Tsiantou *et al.* and Lalonde *et al.*, participants were interested in deeper and more comprehensible information to increase their understanding of their medications, as this had not been offered by their GP’s [15,18]. All participants expressed critical attitudes towards medications, which may be one reason why the ‘second opinion’ from the pharmacist seemed to be of great importance. This ‘second opinion’ had also made them feel comfortable and safe with their medication regime. The pharmacist’s ability to identify inappropriate prescribing and drug-related problems through medication therapy reviews is widely recognized and embraced by the philosophy of *pharmaceutical care*[26-29]. This also corresponds to the participants’ acknowledgement of the pharmacist’s involvement in quality checking medication regimes.

Non-adherence to medications use has been shown to be one of the most important factors for therapy failure [30]. In our study, participants stated that they had become more adherent to their medication regimes as their level of knowledge had increased. They were also interested in taking part in decision making in relation to their medications; not only obeying their doctors. It might seem like adherence with the GP’s prescribing was replaced with the principle of ‘concordance’ during the follow-up program. However, this needs further investigation.

According to our findings, pharmacists should be involved in patient care at different levels; medication information to patients, collaboration with physicians in medication-related questions and quality check of drug regimens (both at hospitals and in community care), and individual medication consultations, for instance at the ‘Heart School’. In general, pharmacists were acknowledged to have more thorough knowledge about medicines compared to physicians. In other countries, for instance in the USA and the UK, pharmacists are already participating in these kind of activities, which has been shown to improve health-related outcomes [31]. It was however surprising that the community pharmacy were disregarded as a place for drug information, and only acknowledged as a place for drug dispensing. This is contradictory to the Norwegian legal framework of the pharmacies, where the role concerning medication information is emphasized [32]. This perception needs further investigation, as this will be crucial for further development of patient-centred community pharmacy services.

Despite a consistent positive attitude to the pharmacist as part of the health-care team, we need to take into account that participants were concerned about how the pharmacist-physician collaboration should function without friction. The fact that pharmacists are not fully acknowledged as part of the interdisciplinary team in Norway today may have contributed to this finding. It will be essential to clarify the clinical pharmacist’s role in the healthcare team, allowing patients to know exactly what to expect from the different health-care personnel.

### Credibility

“*Credibility* deals with the focus of the research and refers to confidence in how well data and processes of analysis address the intended focus” [22]. In our study, several elements need to be considered: i) The number of informants may not have been sufficient to embrace all aspects of the follow-up program and a larger study may have shown more variation in findings. However, the four main topics were explored and elaborated satisfactory by all participants. The results of a qualitative study cannot be generalized in the same way as in quantitative studies. The study has, however, shown what it *may* be like to be in the follow-up program–an insight that in itself may enrich our body of knowledge and also initiate new research questions. ii) The interviews focused on four main topics, which is also reflected in the findings above. The narrative approach was important, and a semi-structured interview-guide was supposed to allow the participants to tell their own story in their own words. We wanted to gain knowledge concerning the main topics, but also concerning other relevant aspects not covered by the main topics. We realize that focus on the main topics may have been too strong to allow for the latter to happen. iii) The interviews were performed at the Department of Pharmacy. Dahlberg argues that interviews *in* the participants’ homes is experienced as safer and more Comfortable [33]. Consequently, we might have gained more knowledge if interviews were performed in the participants’ home. (iv) Verbatim transcribing of the interviews was done in ‘bokmål’, which was neither spoken by the informants nor by the interviewer. This may have introduced misunderstandings. We did try to diminish this by listening to the text several times during transcription. Additionally, quotes have been translated from Norwegian to English, which again may have introduced ambiguity in interpretation. This was sought diminished by verifying translation by several parts. Nevertheless, some degree of interpretation cannot be avoided because qualitative research is a product of *reflexivity* (that fact that the researcher’s pre-understanding and decisions will inevitable have impact upon the meaning and context of the experience under investigation between informants, interviewer and analysing process) [34]. This has to be interpreted by the reader in the light of our descriptions. v) The second analysis was performed by the PI, who was the clinical pharmacist during the follow-up program. Recall bias has been minimized by concealing patient identity and performing the second analysis more than twelve months after the end of the follow-up program. Nevertheless, the analysis may be biased by personal perceptions and negative patient experiences with the follow-up program may be concealed. Because the narratives were analysed twice by different persons and validated by the research group, we have sought to diminish also this bias.

### Dependability

*Dependability* concerns “the degree to which data change over time and alterations made in the researcher’s decisions during the analysis process” [22]. Our data comprised four narratives that were collected during a relative short period of time. The time factor is not considered crucial in relation to the content of the interviews. The interviewer may have changed his interviewing technique slightly from interview to interview, which is considered one of the hallmarks of good qualitative methodology; variability rather than standardization, also called *flexibility*[34]. It is, however, important that *flexibility* is considered alongside *reflexivity*[34]. In this case, that the interviewer was an undergraduate pharmacist student, inexperienced in qualitative interviewing and possibly biased by the fact that he thought this program was positive. If the interviewer for instance had a different professional background, had more experience or was negative towards such a program, results might have been different and other aspects of the program may have been revealed.

### Transferability

*Transferability* refers to “the extent to which the findings can be transferred to other settings or groups” [22]. We only included patients with CHD, who were participants of the follow-up program. Our results consequently reflect our new knowledge concerning this particular patient group and this particular follow-up program. However, as CHD is the number one disease worldwide, and also frequently co-morbid to other diseases, for example diabetes, renal failure and chronic obstructive pulmonary disease (COPD), our findings may be applicable also in other patient groups. However, this remains to be explored. It is said that “the most useful indicator of credibility of the findings is when the practitioners themselves and the readers of the theory view the study findings and regard them as meaningful and applicable in terms of their experience” [35].

## Conclusions

We have through four semi-structured interviews gained knowledge about how post-discharged patients with established CHD experienced a twelve-month lasting follow-up program managed by a clinical pharmacist. The study is limited by the low number of participants. However, the program was highly appreciated, mostly because participants had achieved a higher level of knowledge concerning their medications, but also because their medication regime had undergone a ‘quality check’. Our findings must be seen in connection with the participants’ rather negative experience with their GPs, including shortage of time and dissatisfaction with information, information quality and follow-up. These factors most likely contributed to their increased feeling of safety with the follow-up program. Surprisingly, community pharmacies were not considered a place for medication information by the four participants, which need to be further investigated, as community pharmacies are moving into more patient-centred care.

The clinical pharmacist was well recognized as part of the interdisciplinary healthcare team to be involved in medication information to patients, support for physicians in medication-related questions, and also as independent care givers. It is however important that the role of the clinical pharmacists is clarified, in order for patients to know what to expect from different healthcare takers. We believe our findings are not restricted to patients with CHD only, but may apply also to other patient populations; though further research is needed. Finally, we encourage pharmacists to continue focusing on patient-centred care, and physicians to increase their collaboration with pharmacists in medication-related questions. This seems to be highly appreciated among patients and may be a step towards improving patient care.

## Abbreviations

CHD: Coronary heart disease; CRCC: Cardiac risk reduction clinic; GP: General practitioner; RCT: Randomized controlled trial.

## Competing interests

The authors declare that they have no competing interests.

## Authors’ contributions

BHG had the idea for the study and supported the master student during the whole study in methodological and analytical issues. She is the main writer of the manuscript. SLS and LS both supported the master student in study design and analysis of qualitative data. They have as well been drafting and revising the manuscript, and also been part of translating from Norwegian to English. All authors read and approved the final manuscript.

## References

[B1] Mackay J Mensah G Atlas of Heart Disease and Stroke 2004 Part III, Chapter 14 Deaths from Coronary Heart Disease [http://www.who.int/entity/cardiovascular_diseases/en/cvd_atlas_14_deathHD.pdf] Accessed 2014-03-24

[B2] Kotseva K Wood D The challenge for preventive cardiology Eur J Cardiovasc Prev Rehabil 2009 16 Suppl 2 S19 S23 19675431 10.1097/01.hjr.0000359231.80893.ac

[B3] Kotseva K Treatment of patients with coronary heart disease fails to meet standards of european guidelines: results of EUROASPIRE surveys Rev Esp Cardiol 2009 62 10 1095 1098 10.1016/S0300-8932(09)72377-2 19793514

[B4] WooD DA Kotseva K Connolly S Jennings C Mead A Jones J Holden A De Bacquer D Collier T De Backer G Faergeman O Nurse-coordinated multidisciplinary, family-based cardiovascular disease prevention programme (EUROACTION) for patients with coronary heart disease and asymptomatic individuals at high risk of cardiovascular disease: a paired, cluster-randomised controlled trial Lancet 2008 371 9629 1999 2012 10.1016/S0140-6736(08)60868-5 18555911

[B5] Till LT Voris JC Horst JB Assessment of clinical pharmacist management of lipid-lowering therapy in a primary care setting J Manag Care Pharm 2003 9 3 269 273 14613471 10.18553/jmcp.2003.9.3.269 PMC10437297

[B6] Hunt JS Siemienczuk J Pape G Rozenfeld Y MacKay J LeBlanc BH Touchette D A randomized controlled trial of team-based care: impact of physician-pharmacist collaboration on uncontrolled hypertension J Gen Intern Med 2008 23 12 1966 1972 10.1007/s11606-008-0791-x 18815843 PMC2596500

[B7] Bailey TC Noirot LA Blickensderfer A Rachmiel E Schaiff R Kessels A Braverman A Goldberg A Waterman B Dunagan WC An intervention to improve secondary prevention of coronary heart disease Arch Intern Med 2007 167 6 586 590 10.1001/archinte.167.6.586 17389290

[B8] Axtell SS Ludwig E Lope-Candales P Intervention to improve adherence to ACC/AHA recommended adjunctive medications for the management of patients with an acute myocardial infarction Clin Cardiol 2001 24 2 114 118 10.1002/clc.4960240204 11214740 PMC6654903

[B9] Chapman NR Fotis MA Yarnold PR Gheorghiade M Pharmacist interventions to improve the management of coronary artery disease Am J Health Syst Pharm 2004 61 24 2672 2678 15646702 10.1093/ajhp/61.24.2672

[B10] Horning KK Hoehns JD Doucette WR Adherence to clinical practice guidelines for 7 chronic conditions in long-term-care patients who received pharmacist disease management services versus traditional drug regimen review J Manag Care Pharm 2007 13 1 28 36 17269834 10.18553/jmcp.2007.13.1.28 PMC10438371

[B11] The Framingham Risk Score [http://www.framinghamheartstudy.org/risk-functions/coronary-heart-disease/10-year-risk.php] - Accessed 2014-03-24

[B12] Taveira TH Wu WC Martin OJ Schleinitz MD Friedmann P Sharma SC Pharmacist-led cardiac risk reduction model Prev Cardiol 2006 9 4 202 208 10.1111/j.1520-037X.2006.05339.x 17085982

[B13] Reilly V Cavanagh M The clinical and economic impact of a secondary heart disease prevention clinic jointly implemented by a practice nurse and pharmacist Pharm World Sci 2003 25 6 294 298 14689819 10.1023/b:phar.0000006526.55922.8d

[B14] Lee JK Grace KA Taylor AJ Effect of a pharmacy care program on medication adherence and persistence, blood pressure, and low-density lipoprotein cholesterol: a randomized controlled trial JAMA 2006 296 21 2563 2571 10.1001/jama.296.21.joc60162 17101639

[B15] Lalonde L Hudon E Goudreau J Belanger D Villeneuve J Perreault S Blais L Lamarre D Physician-pharmacist collaborative care in dyslipidemia management: the perception of clinicians and patients Res Social Adm Pharm 2011 7 3 233 245 10.1016/j.sapharm.2010.05.003 21272548

[B16] Lambert-Kerzner A Havranek EP Plomondon ME Albright K Moore A Gryniewicz K Magid D Ho PM Patients' perspectives of a multifaceted intervention with a focus on technology: a qualitative analysis Circ Cardiovasc Qual Outcomes 2010 3 6 668 674 10.1161/CIRCOUTCOMES.110.949800 20923992

[B17] Wheeler A Crump K Lee M Li L Patel A Yang R Zhao J Jensen M Collaborative prescribing: A qualitative exploration of a role for pharmacists in mental health Res Social Adm Pharm 2012 8 3 179 92 10.1016/j.sapharm.2011.04.003 21831724

[B18] Tsiantou V Pantzou P Pavi E Koulierakis G Kyriopoulos J Factors affecting adherence to antihypertensive medication in Greece: results from a qualitative study Patient Prefer Adherence 2010 4 335 343 20859460 10.2147/ppa.s12326 PMC2943225

[B19] Bereznicki B Peterson G Jackson S Haydn WE DeBoos I Hintz P Perceived feasibility of a community pharmacy-based asthma intervention: a qualitative follow-up study J Clin Pharm Ther 2011 36 3 348 355 10.1111/j.1365-2710.2010.01187.x 21545614

[B20] Guerreiro M Cantrill J Martins P Acceptability of community pharmaceutical care in Portugal: a qualitative study J Health Serv Res Pol 2010 15 4 215 222 10.1258/jhsrp.2010.009121 20466750

[B21] Mahmoud HF Farmasøytisk oppfølging av pasienter med hjertesykdom: en kvalitativ studie av pasientenes erfaringer Eng: Pharmaceutical follow-up of patients with coronary heart disease: a qualitative study of patient experiences Masters thesis 2010 University of Tromsoe, Department of Pharmacy [http://munin.uit.no/handle/10037/2754] Accessed 2014-03-24

[B22] Graneheim UH Lundman B Qualitative content analysis in nursing research: concepts, procedures and measures to achieve trustworthiness Nurse Educ Today 2004 24 2 105 112 10.1016/j.nedt.2003.10.001 14769454

[B23] The World Medical Association Declaration of Helsinki - Ethical Principles for Medical Research Involving Human Subjects 2008 Report. [http://wma.net/en/30publications/10policies/b3/] Accessed 2014-03-24

[B24] Horne R Weinman J Barber N Elliot R Concordance, adherence and compliance in medicine taking: report for the National Co-ordinating Centre for NHS Sercive Delivery and Organisation R&D (NCCSDO) 2005 Report. [http://www.academia.edu/855004/Concordance_Adherence_and_Compliance_in_Medicine_Taking]

[B25] Aronson JK Compliance, concordance, adherence Br J Clin Pharmacol 2007 63 4 383 384 10.1111/j.1365-2125.2007.02893.x 17378797 PMC2203247

[B26] Hanlon JT Weinberger M Samsa GP Schmader KE Uttech KM Lewis IK Cowper PA Landsman PB Cohen HJ Feussner JR A randomized, controlled trial of a clinical pharmacist intervention to improve inappropriate prescribing in elderly outpatients with polypharmacy Am J Med 1996 100 4 428 437 10.1016/S0002-9343(97)89519-8 8610730

[B27] Strand LM Cipolle RJ Morley PC Frakes MJ The impact of pharmaceutical care practice on the practitioner and the patient in the ambulatory practice setting: twenty-five years of experience Curr Pharm Des 2004 10 31 3987 4001 10.2174/1381612043382576 15579084

[B28] Viktil KK Blix HS Moger TA Reikvam A Interview of patients by pharmacists contributes significantly to the identification of drug-related problems (DRPs) Pharmacoepidemiol Drug Saf 2006 15 9 667 674 10.1002/pds.1238 16598835

[B29] Hepler CD Strand LM Opportunities and responsibilities in pharmaceutical care Am J Hosp Pharm 1990 47 3 533 543 2316538

[B30] Ho PM Rumsfeld JS Masoudi FA McClure DL Plomondon ME Steiner JF Magid DJ Effect of Medication Nonadherence on Hospitalization and Mortality Among Patients With Diabetes Mellitus Arch Intern Med 2006 166 17 1836 1841 10.1001/archinte.166.17.1836 17000939

[B31] Barnett MJ Frank J Wehring H Newland B VonMuenster S Kumbera P Halterman T Perry PJ Analysis of pharmacist-provided medication therapy management (MTM) services in community pharmacies over 7 years J Manag Care Pharm 2009 15 1 18 31 19125547 10.18553/jmcp.2009.15.1.18 PMC10438125

[B32] LOV 2000-06-02 nr 39: Lov om apotek (apotekloven) [Eng: Norwegian Law concerning Pharmacies of June 2, 2000] Statute [http://lovdata.no/dokument/NL/lov/2000-06-02-39] Accessed 2014-03-24

[B33] Dahlberg K Dahlberg H Nystrøm M An open lifeworld approach Reflective Lifeworld Research 2008 2 Lund: Studentlitteratur 95 121

[B34] Horsburgh D Evaluation of qualitative research J Clin Nurs 2003 12 2 307 312 10.1046/j.1365-2702.2003.00683.x 12603565

[B35] Cutcliffe JR McKenna HP Establishing the credibility of qualitative research findings: the plot thickens J Adv Nurs 1999 30 2 374 380 10.1046/j.1365-2648.1999.01090.x 10457239

